# Index participant characteristics and HIV assisted partner services efficacy in Kenya: results of a cluster randomized trial

**DOI:** 10.1002/jia2.25305

**Published:** 2019-07-19

**Authors:** Sarah J Masyuko, Peter K Cherutich, Marielle G Contesse, Peter M Maingi, Beatrice M Wamuti, Paul M Macharia, David E Bukusi, Felix A Otieno, Hans ML Spiegel, Matthew D Dunbar, Matthew R Golden, Barbra A Richardson, Carey Farquhar

**Affiliations:** ^1^ National AIDS and STI Control Program Ministry of Health Nairobi Kenya; ^2^ Department of Global Health University of Washington Seattle WA USA; ^3^ Department of Preventive and Promotive Health Services Ministry of Health Nairobi Kenya; ^4^ Department of Epidemiology University of Washington Seattle WA USA; ^5^ VCT and HIV Prevention Unit Kenyatta National Hospital Nairobi Kenya; ^6^ Department of Research and Programs Kenyatta National Hospital Nairobi Kenya; ^7^ Department of Health and Human Services Kelly Government Solutions Contractor to National Institute of Allergy and Infectious Diseases National Institutes of Health Rockville MD USA; ^8^ Department of Computer Science and Demography University of Washington Seattle WA USA; ^9^ Department of Medicine University of Washington Seattle WA USA; ^10^ Department of Biostatistics University of Washington Seattle WA USA

**Keywords:** partner services, Africa, HIV prevention, clinical trials

## Abstract

**Introduction:**

We have previously demonstrated that assisted partner services (aPS) increases HIV testing and case finding among partners of persons living with HIV (PLHIV) in a cluster randomized trial in Kenya. However, the efficacy of aPS may vary across populations. In this analysis, we explore differences in aPS efficacy by characteristics of index participants.

**Methods:**

Eighteen HIV testing sites were randomized to immediate versus 6‐week delayed aPS. Participants were PLHIV (or index participants) and their sexual partners. Partners of index participants were contacted for HIV testing and linked to care if HIV positive. Primary outcomes were the number of partners per index participant who: 1) tested for HIV, 2) tested HIV positive and 3) enrolled in HIV care. We used generalized estimating equations to assess differences in aPS efficacy by region, testing location, gender, age and knowledge of HIV status.

**Results:**

From 2013 to 2015, the study enrolled 1119 index participants, 625 of whom were in the immediate group. These index participants named 1286 sexual partners. Immediate aPS was more efficacious than delayed aPS in promoting HIV testing among partners in high compared to low HIV prevalence regions (Nyanza incidence rate ratio (IRR) 7.2; 95% confidence interval (CI) 5.4, 9.6 vs. Nairobi/Central IRR 3.4 95% CI 2.3, 4.8). Higher rates of partner HIV testing were also observed for index participants in rural/peri‐urban compared to urban sites (IRR 6.6; 95% CI 4.5, 9.6 vs. IRR 3.5 95% CI 2.5, 5.0 respectively), for female versus male index participants (IRR 5.8 95% CI 4.2, 7.9 vs. IRR 3.7; 95% CI 2.4, 5.8 respectively) and for newly diagnosed versus known HIV‐positive index participants (IRR 6.0 95% CI 4.2, 8.7 vs. IRR 3.3; 95% CI 2.0, 7.7 respectively). Providing aPS to female versus male index participants also had a significantly higher HIV case finding rate (IRR 9.1; 95% CI 4.0, 20.9 vs. IRR 3.2 95% CI 1.7, 6.0 respectively.)

**Conclusions:**

While it is known that aPS promotes increases in HIV testing and case finding, this is the first study to demonstrate significant differences in aPS efficacy across characteristics of the index participant. Understanding these differences and their drivers will be critical as aPS is brought to scale in order to ensure all PLHIV have access to these services.

## 1 Introduction

Knowledge of one's HIV status is a prerequisite to accessing HIV care and treatment 1, 2. The United Nations global call to control the HIV epidemic, aims to have 90% of people living with HIV (PLHIV) know their status, 90% of those diagnosed on antiretroviral therapy (ART), and 90% of those on ART virally suppressed 3.Globally, three of the four PLHIV knew their status in 2017 4. In 2012, only 47% of Kenyans infected with HIV were aware of their status 5. This has dramatically changed in the past decade due to the tremendous effort and investment in HIV testing and the diversification of testing approaches. Of the 19.6 million PLHIV in eastern and southern Africa in 2017, it is estimated 81% knew their HIV status 4. However, the progress made thus far in HIV testing is largely due to routine HIV testing at the facility and community level and increased use of rapid HIV tests. As countries strive to achieve the first “90,” finding new HIV infected individuals utilizing conventional HIV testing strategies will become increasingly difficult and costly. These challenges highlight the need for more efficient and cost‐effective HIV testing strategies to reach those undiagnosed with HIV.

Assisted partner services (aPS) is a public health strategy where a trained service provider assists PLHIV (“index participants”) who have consented to the service, to disclose their status or to notify and test their named sexual and/or injecting partners 6. This can be offered using a contract, provider or dual referral approach. In contract referral, the trained provider agrees with the index client to disclose their HIV status to their partner(s) within a specific time period. This differs from provider referral where the provider confidentially contacts and notifies the partners of their potential exposure to HIV without waiting for the index to reach out to their partners. APS has been shown to be feasible and effective in newly diagnosing HIV infected individuals in both high‐ and low‐resource settings 7, 8, 9, 10, 11, 12, 13.

This study is a secondary data analysis of a large cluster randomized trial undertaken in Kenya in 2015 that compared immediate and delayed aPS. This cluster randomized trial showed a four to fivefold increase in HIV testing, HIV case‐finding and linkage of HIV‐positive partner to care among recipients of immediate aPS 14. A meta‐analysis conducted by the World Health Organization (WHO) of three randomized trials in Malawi and the United States, showed that aPS increased HIV testing uptake among the partners of index participants and increased the identification of new HIV cases 6, 10, 15, 16, 17. APS has also proven to be feasible, effective and cost‐effective 7, 8, 9, 10, 11, 18, 19, 20, 21, 22, 23, 24, 25.

Following release of WHO guidelines on partner notification in 2016, more countries have included assisted partner notification approaches to existing passive approaches as a strategy to achieve the first 90 on HIV testing 6. As governmental and non‐governmental organizations begin to scale‐up aPS, reaching men, younger and older age groups, key populations, and those living in rural areas may be challenging due to difficulty in accessing and locating sexual partners, but may produce significantly higher HIV case finding as they contribute a large proportion of those unaware of HIV infection. In Kenya and South Africa, for example, undiagnosed HIV infection has been found to be associated with older age, gender, marital status, residence and sexual risk behaviour including multiple sexual partners 5, 26, 27, 28. Although immediate aPS was overall efficacious in diverse populations, the efficacy of aPS in subsets of the populations is not clear. Some studies suggest that this could vary by gender, race and by access to HIV testing 7, 11, 19, 29, 30.

The objective of this study was to assess the efficacy of aPS by the characteristics of index participants defined by region of residence (high vs. medium/low prevalence), rural/peri‐urban versus urban location, gender, age and knowledge of HIV status using data from this Kenya multicentre cluster randomized trial on aPS. Information from this analysis will help identify populations that are underserved with current programming in order to improve access to aPS among all PLHIV in resource limited settings 31, 32.

## 2 Methods

### 2.1 Study design

For this subgroup analysis, we used data from a multicentre, cluster‐randomized controlled trial of aPS in Kenya. The trial methods have been described previously 14, 33. In brief, the study included 18 HIV testing sites selected either from Nyanza or Nairobi/Central region and represented both rural and urban settings. These facilities captured the differences in geography, rural or urban location and HIV prevalence. There were five urban sites, three peri‐urban and one rural site in each arm. Restricted randomization was used to assign the 18 sites (1:1) to immediate aPS and aPS delayed by six weeks and to ensure balance in region and location (urban, peri‐urban and rural).

### 2.2 Study intervention

Nine clinics were randomized to receive immediate assisted partner notification where the partners of an index HIV infected person were immediately notified of HIV exposure by the health provider and offered HIV counselling and testing (immediate aPS). The other nine clinics were randomized to receive delayed assisted partner notification where the index clients were encouraged to notify their sexual partners on their own (delayed aPS). The six week delay provided a control group for those who received immediate notification. This control group provided the passive referral standard of care in Kenya. Further details are provided in the protocol and study paper 33. The study population included HIV infected index participants and their sexual partners.

### 2.3 Eligibility criteria

To be eligible for the study, index participants had to be at least 18 years old, newly diagnosed with HIV at one of the study sites, not enrolled in HIV care, and willing to provide informed consent and information on sexual partners in the preceding three years. Sexual partners had to be at least 18 years old and provide informed consent. This study was limited to the general population. While we collected data on transactional sex were not able to identify key populations.

### 2.4 Study procedures

Clients who tested HIV positive were referred to the health advisors who were certified HIV testing services counsellors additionally trained on aPS. Following informed consent and enrolment into the study, health advisors collected data on demographic characteristics, sexual behaviours, HIV testing history, HIV care and treatment status, economic factors and social harms from index participants. This also included information on each of the participant's sex partners in the preceding three years. In both the immediate and delayed arms, the participants were encouraged to disclose their HIV status to their sexual partners. In the immediate arm, health advisors notified partners within one to two weeks of their potential exposure to HIV and offered HIV testing. Partners in the delayed arm were contacted six weeks after the index participant enrolment. Health advisors offered HIV testing at the study site or at a convenient location and time. Following four failed attempts to contact partners by phone, the health advisors would physically trace the partners.

Human subjects approval was obtained from the University of Washington Institutional Review Board and locally from the Kenyatta National Hospital (KNH)/University of Nairobi (UoN) Ethical and Scientific Review Committee. All participants provided written informed consent. ClinicalTrials.gov registration number is NCT01616420.

### 2.5 Primary outcomes and dependent variables

The primary outcomes were the number of partners per index participant who: 1) tested for HIV, 2) tested HIV positive and 3) enrolled in HIV care following an HIV diagnosis.

A facility was defined as urban, rural or peri‐urban depending on its proximity to a city. Urban areas have higher levels of population density, income and education as compared to rural areas. While peri‐urban areas are in close proximity to urban areas, their socio‐economic profiles are similar to rural areas. We therefore combined peri‐urban and rural sites and compared these to urban sites that have different characteristics. Regions were defined by their county and location in relation to administrative boundaries. HIV prevalence overall is evaluated by region and is presented in Table 1 33. Nairobi, Kiambu and Muranga counties were clustered as Nairobi/Central region with a medium to low HIV prevalence. Kisumu and Siaya County were clustered as Nyanza region and reflect a high HIV prevalence region. Gender was reported as either male or female. Age was dichotomized at the age of 30 years. Participants who had previously tested for HIV prior to study enrolment were categorized as new or known positive depending on the self‐reported result of their last HIV test. New positives are index participants who previously tested for HIV and reported a negative test result in their last test while known positives refers to index participants who previously tested for HIV and reported a positive result. We present the variation in HIV prevalence and ART coverage in Table 1.

**Table 1:** National estimates of HIV prevalence and antiretroviral treatment coverage per county in 2013 [34,37]

Region	County	Location	HIV prevalence^a^	ART coverage^a^
Nairobi	Nairobi	Urban (8)	6.8%	74%
Central	Kiambu	Peri‐urban (2)	5.2%	66%
	Muranga	Peri‐urban (1)	3.8%	32%
Nyanza	Kisumu	Urban (2) and Peri‐urban (3)	19.3%	54%
	Siaya	Rural (2)	23.7%	43%

a Data from the Kenya HIV estimates report 2014.

### 2.6 Statistical analysis

We summarized continuous variables using median and inter‐quartile range (IQR) and categorical variables using percentages. We tested for differences in patient characteristics between regions, location and gender using chi‐square tests for categorical subgroups and a 2‐group independent means *t*‐test for continuous variables. We calculated incidence rate ratios (IRR) using generalized estimating equation (GEE) models with a Poisson link, robust standard errors and exchangeable correlation structure. This model was chosen to account for within clinic correlation. IRRs were calculated for each outcome, stratified by region, location, gender, age and knowledge of HIV status. We tested for interaction between receiving immediate aPS and region, location, gender, age and knowledge of HIV status on the number of partners tested, newly diagnosed, and newly linked to care using a 5% alpha level. All analyses were conducted using Stata version 14.0 (StataCorp, College Station, TX).

## 3 Results

### 3.1 Baseline characteristics

Between August 2013 and August 2015, 1760 index participants were approached to participate in the study, 1183 were assessed for eligibility and 1119 were enrolled. The study enrolled 550 participants from the nine clinics randomized to immediate aPS and 569 participants from the nine clinics randomized to delayed aPS. There were 1286 sexual partners (621 in the immediate arm and 665 in the delayed arm) who enrolled in the study.

Among index participants, the median age was 30 years (IQR 25, 38), 437 (37%) were from Nyanza region, 495 (42%) from rural or peri‐urban areas and 690 (58%) were female. The majority of the index participants were married, employed and had previously been tested for HIV (Tables 2, 3 to 4). The median age of sexual partners was 31 years (IQR 26, 38), with 560 (44%) being females.

**Table 2:** Index participant baseline characteristics, by randomization arm and region

	Nyanza		Nairobi/Central	
	n (%)		n (%)	
	Immediate (N = 256)	Delayed (N = 181)	Immediate (N = 294)	Delayed (N = 388)
Socio‐demographic factors				
Age (years) median (IQR^a^)	29 (25, 35)	28 (24, 36)	30 (25, 39)	32 (27, 39)
Sex (female)	150 (58.6)	108 (59.7)	172 (58.5)	260 (67.0)
Marital status^a^				
Single	45 (17.6)	40 (22.1)	59 (20.1)	60 (15.5)
Married‐monogamous	154 (60.2)	94 (51.9)	172 (58.5)	228 (58.9)
Married‐polygamous	20 (7.8)	21 (11.6)	11 (3.7)	22 (5.7)
Separated/widowed/divorced	37 (14.5)	26 (14.4)	52 (17.7)	78 (20.1)
Employment^b^				
Unemployed	67 (27.1)	44 (25.0)	62 (21.2)	67 (18.1)
Employed	174 (70.5)	130 (73.9)	228 (78.1)	300 (80.7)
Student	6 (2.4)	2 (1.1)	2 (0.7)	5 (1.3)
Sexual behaviour				
Number of lifetime sexual partners Median (IQR)	3 (2.6)	4 (3.6)	4 (3.6)	5 (3.9)
Ever paid money to have sex^a^	64 (25.0)	40 (22.1)	38 (12.9)	47 (12.1)
Ever received money to have sex^a^	63 (24.6)	44 (24.3)	31 (10.5)	78 (20.1)
Ever tested for HIV	172 (67.2)	126 (69.6)	207 (70.4)	240 (61.9)

IQR, interquartile range.

^a^ *p* values are significant for age (<0.001), marital status (0.006), ever paid money to have sex (<0.001), ever received money for sex (0.004); ^b^missing occupation data for nine in Nyanza immediate arm, five in Nyanza delayed arm, two in Nairobi immediate arm and sixteen in Nairobi delayed arm.

**Table 3:** Index participant baseline characteristics, by randomization arm and location

	Urban		Rural	
	N (%)		N (%)	
	Immediate (N = 302)	Delayed (N = 322)	Immediate (N = 248)	Delayed (N = 247)
Socio‐demographic factors				
Age (years) median (IQR)	30 (25, 38)	31 (25, 38)	30 (26, 37)	32 (27, 38)
Sex (female)	187 (62.0)	208 (64.6)	135 (54.4)	160 (64.8)
Marital status^a^				
Single	68 (22.5)	59 (18.3)	36 (14.5)	41 (16.6)
Married‐monogamous	174 (57.6)	175 (54.4)	152 (61.3)	147 (59.5)
Married‐polygamous	9 (3.0)	21 (6.5)	22 (8.9)	22 (8.9)
Separated/Widowed/Divorced	51 (16.9)	67 (20.8)	38 (15.3)	37 (15.0)
Employment^b^				
Unemployed	59 (19.7)	59 (19.3)	70 (29.2)	52 (21.5)
Employed	236 (78.7)	242 (79.1)	166 (69.6)	188 (77.7)
Student	5 (1.7)	5 (1.6)	3 (1.3)	2 (0.8)
Sexual behaviour				
Number of lifetime sexual partners Median (IQR)^a^	4 (2.6)	4 (3.6)	4 (3.6)	5 (3.9)
Ever paid money to have sex^a^	44 (14.6)	42 (13.0)	58 (23.4)	45 (18.2)
Ever received money to have sex^a^	39 (12.9)	56 (17.4)	55 (22.2)	66 (26.7)
Used a condom in last sexual contact	78 (25.8)	67 (20.8)	59 (23.8)	44 (17.8)
Ever tested for HIV^a^	213 (70.5)	229 (71.1)	166 (67.0)	137 (71.1)

IQR, interquartile range.

^a^ *p* values significant for marital status (*p* = 0.003), sexual partners in last three months (*p* < 0.001), lifetime sexual partners (*p* = 0.0023), paid (*p* = 0.0018) or received money for sex (*p* = 0.001) and ever tested for HIV before (*p* = 0.0007); ^b^missing occupation data 2 for Urban immediate, 16 for Urban delayed, nine for Rural immediate and five for Rural delayed.

**Table 4:** Index participant baseline characteristics, by randomization arm and gender

	Females		Males	
	N (%)		N (%)	
	Immediate (N = 322)	Delayed (N = 368)	Immediate (N = 228)	Delayed (N = 201)
Socio‐demographic factors				
Age (years) median (IQR)^a^	27 (23, 32)	29 (25, 35)	35 (30, 42)	36 (30, 42)
Marital status				
Single	81 (25.2)	79 (21.5)	23 (10.1)	21 (10.5)
Married‐monogamous	154 (47.8)	185 (50.3)	172 (75.4)	137 (68.2)
Married‐polygamous	18 (5.6)	28 (7.6)	13 (5.7)	15 (7.5)
Separated/Widowed/Divorced	69 (21.4)	76 (20.7)	20 (8.8)	28 (13.9)
Employment^b^				
Unemployed	96 (30.3)	97 (27.4)	33 (14.9)	14 (7.2)
Employed	215 (67.8)	251 (70.9)	187 (84.2)	179 (92.3)
Student	6 (1.9)	6 (1.7)	2 (0.9)	1 (0.5)
Sexual behaviour				
Number of lifetime sexual partners Median (IQR)^a^	3 (2.4)	4 (3.6)	6 (4.10)	6 (4.10)
Ever paid money to have sex^a^	21 (6.5)	10 (2.7)	81 (35.5)	77 (38.3)
Ever received money to have sex^a^	75 (23.3)	107 (29.1)	19 (8.3)	15 (7.5)
Used a condom in last sexual contact^a^	68 (21.2)	66 (17.9)	69 (30.3)	45 (22.4)
Ever tested for HIV^a^	244 (75.8)	262 (71.2)	135 (59.2)	104 (51.7)

IQR, interquartile range.

^a^ *p* values were significant for age (*p* < 0.001), number of sex partners in the past three months (*p* = 0.0173), lifetime sexual partners (*p* < 0.001), paid money for sex (*p* < 0.001), received money for sex (*p* < 0.001) ever tested for HIV (*p* < 0.001); ^b^missing occupation data for five Females immediate, fourteen Females delayed, seven Males immediate and seven Males delayed.

Of the 1119 index participants enrolled in the study, 745 (67%) had previously tested for HIV. Among those who previously tested for HIV, 205 self‐reported to be HIV positive. Out of the 205 known positives, 186 (91%) were not in HIV care. Following enrolment into the study, 148 (80%) of the 186 participants were successfully linked to care.

### 3.2 Results by region, location, gender, age and knowledge of HIV status

#### 3.2.1 Results by region

We compared testing rates per index from baseline (i.e. without the intervention (delayed arm)) with the testing rates after the intervention (immediate arm).Immediate aPS was associated with a statistically significant sevenfold increase in partner testing in Nyanza as compared to a threefold increase in Nairobi (IRR 7.2; 95% CI 5.4, 9.6 vs. IRR 3.4 95% CI 2.3, 4.8; *p* = 0.001) (Table 5). HIV case finding and linkage rates were not significantly different by region. While immediate aPS increased partner HIV testing from a baseline of 13% to 91% in Nyanza region and from 16% to 54% in Nairobi/Central region, new HIV diagnoses had similar increases from 4% to 21% in Nairobi/Central region and 7% to 29% in Nyanza region (Table 6). There was also a non‐significant increase in linkage to care from 3% to 13% in Nairobi/Central region as compared to an increase from 4% to 13% in Nyanza region (Table 6).

**Table 5:** Effect modification of HIV case finding and incremental number needed to interview (NNTI) by region, location, gender, age and knowledge of HIV status

	IRR (95% CI)	IRR (95% CI)	EM^a^ *p* value
Nairobi/Central		Nyanza	
HIV testing	3.4 (2.3, 4.8)	7.2 (5.4, 9.6)	**0.001**
New HIV testing	18.5 (5.0, 149.9)	27.6 (5.1, 150.3)	0.679
New HIV positive	5.2 (2.4, 11.4)	4.3 (3.3, 5.7)	0.656
Linked to care	4.6 (2.3, 9.2)	3.2 (2.4, 4.3)	0.376
**Rural/Peri‐urban**		**Urban**	
HIV testing	6.6 (4.5, 9.6)	3.5 (2.5, 5.0)	**0.018**
New HIV testing	38.8 (6.4, 235.7)	14.9 (7.0, 31.8)	0.338
New HIV positive	4.3 (2.2, 8.3)	6.0 (3.2, 10.9)	0.474
Linked to care	3.0 (1.8, 4.9)	5.3 (2.8, 10.2)	0.168
**Female index participants**		**Male index participants**	
HIV testing	5.8 (4.2, 7.9)	3.7 (2.4, 5.8)	**0.061**
New HIV testing	‐b	‐b	‐
New HIV positive	9.1 (4.0, 20.9)	3.2 (1.7, 6.0)	**0.039**
Linked to care	5.9 (2.7, 13.0)	2.9 (1.6, 5.1)	0.183
**Age less than 30 years**		**Age 30 and older**	
HIV testing	5.3 (3.7, 7.7)	4.2 (3.1, 5.8)	0.165
New HIV testing	38.6 (5.4, 276.1)	14.4 (5.0, 41.8)	0.446
New HIV positive	5.7 (2.7, 12.2)	4.6 (2.6, 8.2)	0.619
Linked to care	4.9 (2.2, 10.)	3.6 (2.0, 6.4)	0.572
**New positives**		**Known positives**	
HIV testing	6.0 (4.2, 8.7)	3.3 (2.0, 7.7)	**0.014**
New HIV testing	‐	‐	‐
New HIV positive	5.1 (3.0, 8.6)	5.0 (1.9, 13.1)	0.978
Linked to care	4.5 (2.2, 9.2)	4.2 (0.9, 19.5)	0.915

Incremental NNTI is the Incremental Number needed to interview. The bold values are the *p* values that are considered statistically significant.

^a^Effect modification *p* value; ^b^could not calculate IRR and NNTI for all participants previously tested for HIV.

**Table 6:** Rates of HIV testing, case finding and linkage to care by region, location, gender, age and knowledge of HIV status

	Immediate APS		Delayed APS		Immediate APS		Delayed APS	
	N	Rate per index	N	Rate per index	N	Rate per index	N	Rate per index
Nairobi/Central					Nyanza			
HIV testing	141	0.537	62	0.159	162	0.914	22	0.127
New HIV testing	40	0.143	3	0.008	38	0.152	1	0.006
New HIV positive	63	0.214	16	0.041	69	0.285	11	0.066
Linked to care	38	0.129	11	0.028	32	0.125	7	0.039
**Rural**					**Urban**			
HIV testing	153	0.883	33	0.134	150	0.573	51	0.161
New HIV testing	38	0.157	1	0.004	40	0.139	3	0.009
New HIV positive	65	0.278	16	0.065	67	0.222	11	0.037
Linked to care	30	0.121	10	0.040	40	0.132	8	0.025
**Female index participants**					**Male index participants**			
HIV testing	171	0.643	41	0.111	132	0.811	43	0.219
New HIV testing	54	0.177	0	0.000	24	0.105	4	0.020
New HIV positive	63	0.199	8	0.022	69	0.316	19	0.100
Linked to care	31	0.096	6	0.016	39	0.171	12	0.060
**Age less than 30 years**					**Age 30 and older**			
HIV testing	161	0.816	34	0.153	142	0.615	150	0.147
New HIV testing	43	0.169	1	0.044	35	0.127	3	0.009
New HIV positive	64	0.251	9	0.044	68	0.244	18	0.529
Linked to care	34	0.127	6	0.026	36	0.127	12	0.035
**New positives**					**Known positives**			
HIV testing	146	0.751	33	0.125	51	0.563	16	0.172
New HIV testing	35	0.140	2	0.008	12	0.116	0	0.000
New HIV positive	56	0.230	12	0.045	18	0.161	3	0.032
Linked to care	31	0.121	7	0.027	10	0.089	2	0.022

#### 3.2.2 Results by location

There was a statistically significant sevenfold increase in partner HIV testing in rural/peri‐urban sites compared to a fourfold increase in urban sites among index clients who received immediate aPS (IRR 6.6; 95% CI 4.5, 9.6 vs. IRR 3.5 95% CI 2.5, 5.0; *p* = 0.018). Partner HIV testing rates increased from 13% without the intervention to 88% with immediate aPS in rural/peri‐urban areas as compared to an increase from 16% to 57% in urban areas. New HIV diagnosis increased from 7% to 28% in rural/peri‐urban areas and from 4% to 22% in urban areas while linkage to care increased from 4% to 12% and 3% to 13% in rural/peri‐urban areas and urban areas respectively (Table 6). These latter differences were not statistically significant when comparing the rates of HIV case finding and linkage to care.

#### 3.2.3 Results by gender

Immediate aPS was associated with a sixfold increase in partner testing among partners of female index participants compared to a fourfold increase among partners of male index participants trending towards significance (IRR 5.8 95% CI 4.2, 7.9 vs. IRR 3.7; 95% CI 2.4, 5.8; *p* = 0.061). In addition, aPS was significantly associated with a ninefold increase in new diagnosis among partners of female index participants as compared to a threefold increase among partners of male index participants (IRR 9.1; 95% CI 4.0, 20.9 vs. IRR 3.2 95% CI 1.7, 6.0; *p* = 0.039). Immediate aPS increased HIV testing from a baseline of 11% to 64% among partners of female index participants and from 22% to 81% among partners of male index participants. New HIV diagnosis increased from 2% without aPS to 20% with immediate aPS among partners of female index participants and 1% to 32% among partners of male index participants (Table 6). There was an increase in linkage to care rates from 2% to 10% among partners of female index participants as compared to an increase from 6% to 17% among partners of male index participants (Table 6).

#### 3.2.4 Results by age

Among index clients who received immediate aPS, there were higher rates of HIV testing for partners of those less than 30 years compared to those who were 30 years and older (Table 5) (IRR 5.3; 95% CI 3.7, 7.7 vs. IRR 4.2 95% CI 3.1, 5.8; *p* = 0.165). While these differences were not statistically significant, HIV testing among partners of index participants aged less than 30 years increased from 15% without aPS to 81% with aPS as compared to an increase from 15% to 62% among partners of index participants aged 30 years and above. We also observed non‐significant changes in HIV case finding and linkage (Table 6).

#### 3.2.5 Results by knowledge of HIV status

Among index participants receiving immediate aPS, there was a statistically significant sixfold increase in HIV testing among partners of index participants who were newly diagnosed with HIV compared to a threefold increase in partners of index participants who were known to be HIV positive (*p* = 0.014). HIV testing increased from 13% without the intervention to 75% with immediate aPS among partners of those newly diagnosed with HIV compared to an increase from 17% to 56% in partners of those known to be positive. HIV case finding increased from 5% to 23% and 3% to 16% among partners of those newly diagnosed with HIV and those known to be HIV positive respectively. Linkage of care increased from 3% to 12% among partners of newly diagnosed index participants as compared to an increase of 2% to 9% among partners of those known to be HIV positive (Table 6).

## 4 Discussion

Previous results for this cluster randomized trial showed that immediate aPS was overall more efficacious than delayed aPS in increasing HIV testing, diagnosing new HIV cases, and linking PLHIV to treatment services. With this paper, we demonstrate that aPS efficacy varies across key index characteristics and achieves higher HIV testing rates for some hard‐to‐reach populations, such as men in rural areas. Specifically, we found rates of HIV testing were significantly higher when offered to index participants in the high HIV prevalence Nyanza region versus lower prevalence regions in Kenya, in rural or peri‐urban facilities rather than urban facilities. HIV testing rates were also higher when offered to female rather than male index participants, and to those who were newly diagnosed with HIV compared to those with known HIV. HIV case finding rates were significantly higher only for female index compared to male index participants. Few HIV partner services studies have conducted analyses by index characteristics; we believe this is the only randomized study with a large sample size that presents an in‐depth analysis of these factors. Our study shows that aPS can be used to reach those who have remained undiagnosed in the setting of existing HIV testing services but there is variability in how efficacious it is when it comes to HIV testing and HIV case finding. This will be critical information for scaling up aPS to different populations, optimizing implementation and understanding outcome variability. APS can also be a useful tool to reengage people known to be HIV positive in care regardless of index characteristics. Our study found that 17% of index participants had been previously diagnosed as HIV positive but were not currently enrolled in care. Of these, we were able to link 80% to ART.

Our findings regarding index location, gender and new HIV infection status can be attributed in large part to variability in HIV testing rates for different populations. Increased efficacy of aPS among female index participants results in part from lower baseline testing, HIV case finding and linkage rates among male partners of female index participants. A smaller percentage of men in Kenya know their HIV status due to fewer testing opportunities and hence when aPS is offered to female index participants, it is more effective in reaching men, closing the testing gap and diagnosing a new HIV infection 35, 36. Since a higher percentage of females know their HIV status through prevention of mother‐to‐child transmission programmes (PMTCT), it is more difficult to find female partners of male index participants who have not been tested and have undiagnosed HIV infection. Our study findings are in line with other studies in Africa and in the United States reporting a higher case finding among female index participants 19, 29. Nonetheless aPS among male index participants was also highly successful. Furthermore, the success in newly diagnosing HIV with aPS remains higher than other testing approaches even if offered to all index participants regardless of gender. We posit that low HIV testing rates in rural areas could also explain the higher efficacy of immediate aPS in rural/peri‐urban areas. This is supported by findings of lower access to testing sites in rural areas in the recent population based health and impact assessments in Africa 35, 36, and higher rates of stigma and discrimination which have been shown to contribute to lower HIV testing uptake in rural and peri‐urban areas 38. Higher aPS efficacy for index who were newly diagnosed HIV positive versus known PLHIV could be explained by different testing rates among their partners. A person living with chronic HIV, who had dropped out of care and is now re‐engaging through repeat testing, might have already disclosed to partners who would have tested. This is an important group of partners to target since a person who does not know his or her status would not have notified partners and might engage in unsafe sexual or injection behaviours, thus increasing risk of transmission 39. Together these data support mapping of areas and populations with low testing rates and high HIV prevalence by health policy planners as a tool for setting more granular aPS targets and identifying areas where aPS would be especially impactful.

Implementing aPS together with other HIV testing strategies that are proven to be effective, such as the secondary distribution of HIV self‐tests to partners of female index participants, may further increase HIV case finding in hard‐to‐reach populations 40. While immediate aPS may reduce the testing gap among men and those in rural/peri‐urban areas, access to services needs to be improved to ensure effective treatment and HIV viral suppression. Interventions to reduce stigma and promote positive attitudes among health providers may also be necessary to increase acceptability and improve delivery of aPS.

Strengths of this study include that data were analysed from a large cluster‐randomized controlled trial with testing sites that were randomized. Our study has several limitations. Participants were consented at baseline and partner contact information was elicited in both the immediate and delayed arm. Elicitation of partner information could have made the participants in the delayed arm more motivated to notify their partners than in normal settings. While this would have resulted in attenuation of the effect, it would not have changed the validity of our results. In addition, this study did not identify index participants who inject drugs and hence we cannot draw conclusions about the efficacy of aPS in this population.

## 5 Conclusions

Our study findings support the WHO guidelines that assisted partner notification is effective and should be provided to all persons with newly diagnosed HIV infection. Understanding characteristics of index participants will enable countries and donors to set realistic targets and develop appropriate strategies for aPS scale‐up, monitoring and evaluation. Widespread implementation targeting male partners may effectively reduce the gap in HIV testing, diagnosis and linkage to care gap between men and women. However, to ensure maximum impact of aPS, access to HIV treatment and prevention services needs to be improved for these populations.

## Competing interests

We declare no conflicts of interest.

## Authors’ contributions

C.F., P.C., M.G., D.B., A.N., H.S., P.M., P.M., M.D. and B.R. developed and implemented the APS trial protocol. S.M., P.C. and C.F. designed this analysis. B.W. and F.O. coordinated data collection. S.M., M.G. and B.R. analysed the data, and S.M. drafted the manuscript. All authors contributed to editing of the manuscript and approved submission of the final draft for publication.
